# Unsupervised clustering reveals phenotypes of AKI in ICU COVID-19 patients

**DOI:** 10.3389/fmed.2022.980160

**Published:** 2022-10-05

**Authors:** David Legouis, Gilles Criton, Benjamin Assouline, Christophe Le Terrier, Sebastian Sgardello, Jérôme Pugin, Elisa Marchi, Frédéric Sangla

**Affiliations:** ^1^Division of Intensive Care, Department of Acute Medicine, University Hospital of Geneva, Geneva, Switzerland; ^2^Laboratory of Nephrology, Department of Medicine and Cell Physiology, University Hospital of Geneva, Geneva, Switzerland; ^3^Geneva School of Economics and Management, University of Geneva, Geneva, Switzerland; ^4^Department of Surgery, Center Hospitalier du Valais Romand, Sion, Switzerland

**Keywords:** AKI, clustering, machine learning, COVID-19, critical care

## Abstract

**Background:**

Acute Kidney Injury (AKI) is a very frequent condition, occurring in about one in three patients admitted to an intensive care unit (ICU). AKI is a syndrome defined as a sudden decrease in glomerular filtration rate. However, this unified definition does not reflect the various mechanisms involved in AKI pathophysiology, each with its own characteristics and sensitivity to therapy. In this study, we aimed at developing an innovative machine learning based method able to subphenotype AKI according to its pattern of risk factors.

**Methods:**

We adopted a three-step pipeline of analyses. First, we looked for factors associated with AKI using a generalized additive model. Second, we calculated the importance of each identified AKI related factor in the estimated AKI risk to find the main risk factor for AKI, at the single patient level. Lastly, we clusterized AKI patients according to their profile of risk factors and compared the clinical characteristics and outcome of every cluster. We applied this method to a cohort of severe COVID-19 patients hospitalized in the ICU of the Geneva University Hospitals.

**Results:**

Among the 248 patients analyzed, we found 7 factors associated with AKI development. Using the individual expression of these factors, we identified three groups of AKI patients, based on the use of Lopinavir/Ritonavir, baseline eGFR, use of dexamethasone and AKI severity. The three clusters expressed distinct characteristics in terms of AKI severity and recovery, metabolic patterns and hospital mortality.

**Conclusion:**

We propose here a new method to phenotype AKI patients according to their most important individual risk factors for AKI development. When applied to an ICU cohort of COVID-19 patients, we were able to differentiate three groups of patients. Each expressed specific AKI characteristics and outcomes, which probably reflect a distinct pathophysiology.

## Introduction

Acute Kidney Injury (AKI) is a common condition in the critical care setting (1, 2). Despite decades of research, AKI is still associated with high mortality and morbidity, even when renal function is substituted by Renal Replacement Therapy (RRT) (3–6).

AKI is defined as a sudden decrease in glomerular filtration rate, demonstrated by an increase in serum creatinine (7). This unified definition has resulted in improved recognition of AKI and has simplified research, healthcare management as well as comparisons across cohorts and different centers. However, AKI is not a single clinical entity but an overarching clinical syndrome. Therefore, the definition of AKI encompasses many underlying conditions and etiologies. Additionally, the high degree of heterogeneity of the Intensive Care Unit (ICU) population including patients with different risk profiles adds further complexity when considering AKI outcomes (8). In this respect, recognizing meaningful subgroups of AKI patients may provide a deeper insight into AKI pathophysiology and may also be helpful in identifying groups with differing prognoses and sensitivity to therapy (9).

From a data-driven perspective, patient sub-phenotyping is essentially a clustering problem (10, 11). Clustering algorithms are a type of unsupervised machine learning algorithms where no labels are known a priori but rather, get assigned based on inherent similarities between points. A critical step in clustering is data representation i.e., the construction of the dataset on which we want to apply clustering. Previous studies on AKI sub-phenotyping have defined patients according to diagnostic codes (12), trajectories of serum creatinine (13), patterns of AKI reversal (14) or clinical and biological data recorded at ICU admission (15) or during AKI (16, 17). However, these strategies do not allow for the formulation of any hypothesis based on the pathophysiological mechanisms involved in different AKI phenotypes. In addition, the high number of features used to classify patients makes it Difficult, in Current Practice, to Recognize Them at the Bedside.

In this study, we aimed to develop an innovative pipeline of analyses in order to identify in an unsupervised manner, distinct phenotypes of AKI in ICU COVID-19 patients based on their pattern of AKI associated factors.

## Materials and methods

### Study design

We conducted a retrospective, single-center, cohort study aiming at identifying factors linked to the development of AKI in order to further clusterize AKI patients according to their pattern of risk factors.

### Patient inclusion

During the study period from March to December 2020, all COVID-19 patients admitted to the adult ICU of the Geneva University Hospitals were screened. Patients were included if they were older than 18 years of age and not on chronic dialysis. They were not included if they experienced an episode of AKI prior to ICU admission, during the same hospital stay. The study was conducted according to the guidelines of the Declaration of Helsinki and approved by the ethical committee for human studies of Geneva, Switzerland (CCER 2020-00917, Commission Cantonale d'Ethique de la Recherche).

### Definitions

AKI was defined according to the serum creatinine based KDIGO criteria (7), i.e., a 1.5-fold or more increase in baseline serum creatinine levels within 7 days or an absolute increase higher than 26.4 μmol/L within 48 h. Baseline serum creatinine levels were determined as the first serum creatinine level recorded following hospital admission. The urine output was not used to identify AKI as it was not recorded for all patients.

### Data collection

For each patient, the following variables were recorded: demographic data (sex, age, body mass index, height, and weight), prior history of hypertension, diabetes, Chronic Obstructive Pulmonary Disease (COPD), hypercholesterolemia, tobacco consumption, cardiomyopathy and heart failure, cerebrovascular disease, malignancy, chronic kidney disease (defined as a history of chronic renal disease in the patient's medical records), chronic use of Non-Steroidal Anti Inflammatory Drugs (NSAIDs), renin angiotensin aldosterone system inhibitors or steroids. Upon ICU admission, we recorded biological data (prothrombin ratio, procalcitonin, C-reactive protein, d-dimer, white blood cells, lymphocytes, neutrophils, thrombocytes, lactate, bilirubin, alanine transaminase (TGP), aspartate transaminase (TGO), troponin levels, serum creatinine and eGFR), severity scores (APACHE, SAPS, SOFA) and the FiO2. Once patients were intubated, we recorded the initial respiratory parameters (PaO2/FiO2 ratio, PEEP and plateau pressure levels, compliance, tidal volume, duration from symptom onset or hospitalization to intubation, respiratory rate before intubation) and the specific therapeutic against COVID-19 (Lopinavir/Ritonavir (LPV/r), hydroxychloroquine, azithromycin, remdesivir, anakinra, dexamethasone). Finally, we screened the following variables for the entire ICU stay: the need for invasive mechanical ventilation, Neuro Muscular Blocking Agents (NMBA), Extra Corporeal Membrane Oxygenation (ECMO), norepinephrine, antibiotics and their total duration, the need for prone positioning and the number of prone sessions, the use of inhaled nitric oxide. At the renal level, we collected all the serum creatinine values recorded during the hospital stay, as well as the need for renal replacement therapy. We also recorded the time between symptoms and admission to hospital, ICU and intubation, the duration between hospital and ICU admission and intubation. Glucose and lactate levels measured during the ICU stay were also collected.

### Metabolic pattern

Five metabolic patterns were defined according to glucose and lactate levels, as previously described (18, 19): the baseline profile (lactate levels below median and with glucose levels between the 25th and the 50th percentile); the impaired metabolism profile (lactate levels above the median with glucose level below the 75th percentile); the isolated hyperglycaemia profile (lactate levels below median with glucose levels above the 75th percentile); the isolated hypoglycaemia profile (lactate levels below median with glucose levels below the 25th percentile) and the stress response profile (lactate levels above median and glucose levels above the 75th percentile). For each patient, we also calculated the relative time spent in one of the five metabolic patterns, i.e., the total duration spent in each of the five profiles divided by the total duration of ICU stay. Finally, the pattern in which the patient spent the most time was considered to be the individual patient metabolic pattern. These five metabolic profiles are shown in Supplementary Figure 1.

### Clinical outcomes

We compared the following outcomes among clusters: AKI severity and recovery, metabolic pattern, and hospital mortality.

AKI severity was determined using KDIGO criteria, while stage 3 was divided into two stages depending on the need of RRT. AKI recovery was defined as serum creatinine levels 1.5 times below the baseline level and the absence for renal replacement therapy following an episode of AKI (20).

### Statistical analysis

Baseline characteristics were expressed as mean (standard deviation) and median (25–75th percentiles) or absolute and relative (%) frequency if categorical. They were compared using a Mann Whitney or Chi-square tests depending on their class. A *p*-value of <0.05 was considered significant

All the analyses were performed using R software (21).

### Pipeline of analyses

Step 1: Identification of AKI associated factors

We began by preprocessing the data by following three steps. First, numerical variables were centered, scaled and normalized through a Yeo-Johnson transformation, because independent variables were on very different scales. This also allowed us to enhance variable selection robustness (22). Supplementary Figure 2 shows the distribution of the numerical variables before and after treatment. Second, we imputed missing data using bagged tree imputation (23) to improve accuracy of downstream analyses (24). Missing data and their distribution for each variable before and after the imputation are presented in Supplementary Figure 3. Third, we calculated a correlation matrix to identify colinear variables, and removed or merged those with a correlation coefficient above 0.8 (Supplementary Figure 4). This step was completed using the *caret* package.

To identify factors associated with AKI development from this pre-processed data, we first looked for variables that fulfilled three criteria: (1) they should exhibit an *a priori* association with AKI, (2) they should be easy to identify by clinicians or be modifiable factors (i.e., therapeutic initiated before AKI onset) and (3) they should be prior to the AKI onset. For this purpose, we considered past medical history including: hypertension, diabetes, Chronic Obstructive Pulmonary Disease (COPD), hypercholesterolemia, tobacco consumption, cardiomyopathy and heart failure, cerebrovascular disease, malignancy, chronic kidney disease and the eGFR at hospital entrance, chronic medication (NSAIDs, renin angiotensin aldosterone system inhibitors or steroids), the demographic data (age, sex, and BMI), the markers of severity at ICU admission (APACHE, SOFA and SAPS scores, FiO2, PaO2/FiO2 ratio), the use of mechanical ventilation and the initiation of COVID-19 specific therapy, started either before or at ICU admission (LPV/r, hydroxychloroquine, azithromycin, remdesivir, anakinra, dexamethasone).

For each, we fitted a univariable logistic spline regression modeling the logit of AKI. Natural restricted cubic splines with two degrees of freedom were used as nonlinear relations between AKI and frequently reported risk factors (25–30).

Variables displaying a *p*-value below 0.2 were considered for the multivariable analyses, which were conducted using a generalized additive model to allow nonlinear relationships via thin plate regression splines (*mgcv* package). Variable selection was further performed using a supervised stepwise approach as previously described, in order to only keep predictors with a *p*-value lower than 0.05 (31, 32). An exception was made for the APACHE score to ensure our model was adjusted for severity. Discrimination and calibration of the final model were visually assessed through the receiver operating characteristic (ROC) curve and a calibration plot as well as numerically by calculating the area under the ROC curve and the Hosmer-Lemeshow test.

The final model was validated as following: Validation of the nonlinear fitting was achieved by building a second generalized additive model. Instead of regression splines, local regression was fitted by locally estimating scatterplot smoothing curve fitting, as supported by the *gam* package. The two nonlinear fits were further visually compared by displaying the partial dependence plots of each model. Validation of the supervised variable selection was performed *via* an unsupervised approach. Three machine learning methods [multistep adaptative MCPnet (MSAMNET), lasso regression and regularized random forest (RRF)], that integrate native automated feature selection, were applied to the dataset. The input matrix of explanatory variables includes all the variables selected in the previous paragraph, i.e., those that fulfilled our three criteria. These three algorithms were applied to the whole dataset, without splitting. A hyperparameter grid was used to tune each model whose performance was iteratively assessed by the out-of-bag area under the ROC curve through a repeated cross-validation procedure (5 repetitions of 10 cross-validations). The selected features and their relative importance were extracted and calculated, for each model, using the varImp command from the *caret* package.

Step 2: Identification of AKI phenotypes

In this second part, we aimed at defining clusters of patients according to the pattern of risk factors expressed by each patient. We started by estimating the relative contribution of each factor identified by the final gam model to the predicted probability of AKI. For this purpose, we calculated the Shapley Additive Explanation (SHAP) values with the *shapr* package using an empirical approach. SHAP values represent a feature's role in changing the model output. The resulting matrix of SHAP values, restricted to AKI patients, was further used as an input for Uniform Manifold Approximation and Projection (UMAP), using a Euclidean metric, a minimal distance of 0.1 and 15 neighbors with the *umap* package. Patients projected on this UMAP were finally clusterized using an unsupervised method: the Density-Based Spatial Clustering of Application with Noise (DBSCAN) algorithm, through the *dbscan* package. The radius of the epsilon neighborhood was set to 1. This 2-step dimensional reduction procedure was adopted to clusterize patients according to their risk profiles and to improve downstream computational clustering (33).

The clustering was further validated by linear support vector machines (SVM, *caret* package) as previously described (34, 35). SVM models were applied to each previously found cluster, to assess its ability to separate this cluster of interest from the others by a hyperplane. For this reason, the UMAP low dimension matrix was first randomly split in a train and a test dataset using a 0.8:0.2 ratio. SVM models were first trained on test dataset, in order to tune their hyperparameters to maximize the area under the ROC curve using repeated cross validation as the resampling method (3 repetitions of 10 cross-validations). The optimal SVM models were further applied to the 2000-fold bootstrapped test datasets.

Step 3: clinical comparisons of the clusters

Subsequently, we compared the identified clusters from a clinical perspective.

For AKI severity, metabolic pattern and hospital mortality, posteriori probability of each outcome in each cluster was calculated using a Naïve Bayes algorithm. Confidence intervals and *p*-values were further estimated through bootstrap resampling (*n* = 2000).

For AKI recovery and hospital mortality, comparisons between clusters were also completed through a Cox Proportional-Hazards Model.

## Results

### Cohort description

From March to December 2020, 253 COVID-19 patients were admitted to the ICU of the Geneva University Hospitals. Among them, 5 were not included because they were on chronic dialysis. A total of 248 patients were analyzed of which 99 (40%) developed AKI. Most of them developed KDIGO1 AKI (67%) while 14 (14%) received Renal Replacement Therapy (RRT). AKI occurred within 3 IQR (1.0–6.0) days following ICU admission. Compared to those who did not develop AKI, AKI patients more frequently reported a history of diabetes and hypertension. They had a lower estimated Glomerular Filtration Rate (eGFR) at hospital entry, were older and mostly male. Furthermore, they had higher APACHE and SOFA scores as well as troponin, C reactive protein and procalcitonin levels but lower bicarbonate levels at ICU admission. AKI patients were more likely to receive norepinephrine, Lopinavir/Ritonavir (LPV/r), hydroxychloroquine and azithromycin but not dexamethasone. Finally, AKI patients more frequently required invasive mechanical ventilation and prone positioning, received higher tidal volumes, spent more time on mechanical ventilation and had longer ICU and hospital lengths of stay. However, mortality was not different between AKI and non-AKI patients. Table 1 compares these characteristics between the two groups.

**Table 1:** Baseline characteristics: Data are presented as mean (percentage) or as median (interquartile range).

	**No-AKI (*N* = 149)**	**AKI (*N* = 99)**	**Total (*N* = 248)**	***P* value**
**Patients' characteristics**				
Age (years),	63.0 (55.0, 73.0)	67.0 (59.0, 74.0)	65.5 (57.0, 74.0)	0.027
Sex, male, *n* (%)	103 (69.1)	84 (84.8)	187 (75.4)	0.006
Weight (cm)	80.0 (70.0, 95.0)	85.0 (73.5, 100.0)	83.3 (70.9, 98.0)	0.061
BMI (mkg/m^2^)	27.8 (24.6, 32.0)	28.3 (25.4, 32.8)	27.8 (24.9, 32.3)	0.137
Hypertension, *n* (%)	59 (39.6)	55 (55.6)	114 (46)	0.019
Diabetes, *n* (%)	38 (25.5)	41 (41.4)	79 (31.9)	0.012
Chronic kidney disease, *n* (%)	6 (4.0)	14 (14.1)	20 (8.0)	0.007
**At ICU admission**				
time from hospital entrance (d)	2.0 (0.0, 5.0)	2.0 (1.0, 4.0)	2.0 (0.0, 5.0)	0.925
eGFR (mL/min/1.73 m^2^)	67.0 (51.8, 91.8)	49.3 (33.8, 66.0)	59.5 (43.4, 83.4)	<0.001
Urea (mmo/L)	6.9 (5.0, 9.4)	7.0 (5.6, 10.3)	7.0 (5.3, 9.6)	0.486
SAPS II score	52.0 (35.0, 65.0)	55.0 (40.5, 66.0)	53.0 (36.0, 65.2)	0.193
APACHE II score	22.0 (13.0, 28.0)	23.0 (15.0, 30.0)	22.0 (14.0, 29.0)	0.048
SOFA score	6 (4.0, 7.0)	6.0 (4.0, 8.0)	6.0 (4.0, 7.0)	0.025
Hemoglobine (g/L)	129.0 (117.0, 144.0)	130.0 (116.5, 143.0)	130.0 (116.8, 144.0)	0.842
Procalcitonin (μg/l)	0.3 (0.2, 0.8)	0.7 (0.3, 2.0)	0.4 (0.2, 1.4)	<0.001
CRP (mg/l)	128.8 (82.3, 193.5)	161.4 (112.5, 216.3)	141.0 (87.5, 205.5)	0.026
White blood cells (G/l)	9.5 (6.3, 13.1)	9.4 (6.5, 11.3)	9.5 (6.4, 12.6)	0.706
Lactate (mmol/l)	1.2 (0.9, 1.7)	1.0 (0.8, 1.5)	1.1 (0.8, 1.7)	0.063
Bilirubin (μmol/l)	9.0 (6.0, 13.0)	9.5 (7.0, 16.0)	9.0 (6.0, 14.0)	0.148
chlore (mmol/l)	104.0 (100.0, 106.0)	104.0 (101.0, 107.0)	104.0 (100.0, 106.0)	0.499
bicarbonates (mmol/l)	25.4 (23.5, 27.0)	23.7 (21.8, 26.3)	24.9 (22.7, 26.7)	<0.001
FiO2 (%)	60.0 (50.0, 80.0)	61.0 (50.0, 80.0)	60.0 (50.0, 80.0)	0.891
P/F	16.4 (11.8, 21.4)	15.0 (11.9, 20.0)	16.0 (11.8, 20.7)	0.416
Tidal volume (mL)	450.0 (400.0, 480.0)	460.0 (430.0, 490.0)	450.0 (420.0, 480.0)	0.015
LPV/r, *n* (%)	16 (11.0)	33 (31.7)	49 (19.6)	<0.001
Azithromycin, *n* (%)	52 (34.9)	57 (57.6)	109 (44.0)	<0.001
Hydroxychloroquine, *n* (%)	54 (36.2)	57 (57.6)	111 (44.8)	0.001
Anakinra, *n* (%)	9 (6.0)	5 (5.1)	14 (5.6)	1.000
Dexamethasone, *n* (%)	84 (56.4)	32 (32.3)	116 (46.8)	<0.001
NSAID, *n* (%)	13 (8.7)	16 (16.2)	29 (11.7)	0.105
Remdesivir, *n* (%)	19 (12.8)	9 (9.1)	28 (11.3)	0.419
Steroids, *n* (%)	49 (32.9)	44 (44.4)	93 (37.5)	0.082
Antibiotics, *n* (%)	142 (95.3)	98 (99.0)	240 (96.8)	0.150
NMBA, *n* (%)	94 (63.1)	84 (84.8)	178 (71.8)	<0.001
Noradrenaline, *n* (%)	115 (77.2)	95 (96.0)	210 (84.7)	<0.001
**During ICU stay**				
Max Serum Creatinine (μmol/l)	81.0 (68.0, 99.0)	163.0 (121.5, 278.5)	100.0 (75.8, 150.5)	<0.001
KDIGO stage, *n* (%)				<0.001
KDIGO1	0 (0.0)	66 (66.7)	66 (26.6)	
KDIGO2	0 (0.0)	13 (13.1)	13 (5.2)	
KDIGO3 without RRT	0 (0.0)	6 (6.1)	6 (2.4)	
KDIGO3 with RRT	0 (0.0)	14 (14.1)	14 (5.6)	
Mechanical ventilation, *n* (%)	121 (81.2)	97 (98.0)	218 (87.9)	<0.001
Prone positioning, *n* (%)	93 (62.4)	79 (79.8)	172 (69.4)	0.005
ECMO, *n* (%)	8 (5.4)	8 (8.1)	16 (6.5)	0.435
**Outcomes**				
ICU mortality, *n* (%)	36 (24.2)	32 (32.3)	68 (27.4)	0.191
Hospital mortality, *n* (%)	37 (24.8%)	32 (32.3%)	69 (27.8%)	0.247
ICU LOS (d),	11.0 (6.0, 18.0)	17.0 (12.0, 23.0)	13.0 (8.0, 21.0)	<0.001
Hospital LOS (d)	25 (16.0, 33.0)	32.0 (23.0, 46.0)	27.0 (16.8, 40.0)	<0.001
Aki recovery, *n* (%)	0 (0.0%)	76 (76.8%)	76 (30.6%)	<0.001

BMI, Body Mass Index; eGFR, estimated glomerular filtration rate; FiO2, Oxygen inspirited fraction; SAPSII, Simplified Acute Physiological Score; APACHE II, Acute Physiology and Chronic Health Evaluation; SOFA, Sepsis-Related Organ Failure Assessment; RRT, Continuous Renal Replacement Therapy; LPV/r, Lopinavir/Ritonavir; NSAID, Non-Steroidal Anti Inflammatory Drug; NMBA, Neuro Muscular Blocking Agents; ECMO, Extra Corporeal Membrane Oxygenation; P/F PaO2/FiO2, ratio; ICU Intensive Care Unit; AKI, Acute Kidney Injury; LOS, Length of Stay.

### Development of a pipeline of analyses

To identify subgroups of AKI patients, we based our approach on unsupervised clustering. However, unlike in previous studies, we did not apply a clustering algorithm on the raw dataset but rather designed a three-step pipeline of analyses. Firstly, we built a nonlinear statistical model to identify factors significantly associated with AKI development in ICU patients and calculated the importance of each predictor for AKI risk at a single patient level. Second, we used unsupervised clustering to identify patterns of AKI-associated factors. Third, we compared the clinical outcomes between those clusters of AKI patients. These three steps are detailed in the methods section.

### Identification of AKI associated factors

#### Explicative statistical model

We first aimed at identifying factors associated with AKI development in COVID-19 patients admitted to the ICU.

The final multivariable model identified 7 variables, which were significantly associated with AKI development in the ICU (Supplementary Table 1): use of LPV/r initiated before ICU admission, diabetes mellitus and invasive mechanical ventilation at ICU admission, were all positively associated with AKI while administration of dexamethasone at ICU admission was protective. APACHE score and FiO2 at ICU admission as well as eGFR at hospital entrance displayed a nonlinear association with AKI.

**Figure 1:**
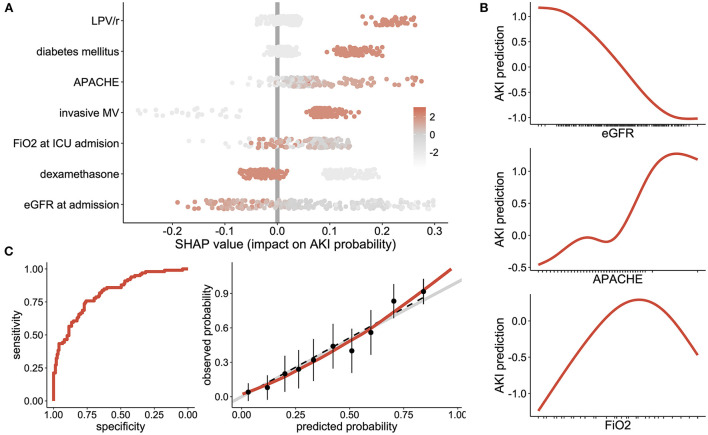
AKI associated factors: **(A)** Shapley Additive Explanation (SHAP) values, where one dot represents the importance of each variable for AKI risk at the single patient level. Positive values reflect an increased risk of AKI while negative values show a negative effect on AKI risk. The sum of all SHAP values from one patient represent the predicted AKI probability for this patient. Each dot is color coded according to the patient's initial value for each considered feature. **(B)** Partial dependence plots, showing the effect of eGFR, APACHE score and FiO2 at ICU admission on the risk of AKI. **(C)** evaluation of the generalized additive model with the receiver operating characteristic curve (left panel) and the calibration plot (right panel) showing sensitivity according to the specificity and the observed vs. predicted probabilities, respectively. LPV/r Lopinavir/Ritonavir, DM Diabetes Mellitus; DXM Dexamethasone; MV Mechanical Ventilation.

Figure 1A displays the SHAP value (x-axis) for each predictor and each patient, while the color of the dot refers to the original value taken by the variable for each patient being considered. The sum of each patient's SHAP values refers to the predicted AKI probability for this patient. Seeing as the relationship between AKI probability and numerical variables was nonlinear, their marginal effect was shown in Figure 1B.

Altogether, the final generalized additive model was discriminant in predicting an AKI ROC curve equal to 0.82 (95% confidence interval [0.77-0.87]), which was well calibrated (*p*-value of the Hosmer–Lemeshow test equal to 0.88), Figure 1C.

#### Sensitivity analyses

A similar non-linear relationship between the risk of AKI and baseline eGFR, tidal volume, FiO2 and APACHE score level at ICU admission was observed in the validation model using a local regression by locally estimated scatterplot smoothing curve fitting instead of regression splines (Supplementary Figure 5A).

In addition, MSAMNET, Lasso and RRF machine learning algorithms ensured the robustness of the variable selection by identifying the following factors: use of dexamethasone, LPV/r, eGFR at hospital admission, invasive mechanical ventilation and prior history of diabetes. These were chosen for every method, while APACHE scores and FiO2 at admission were only captured by the nonlinear method (RRF). Supplementary Figure 5B shows the distribution of the out-of-bag area under the ROC curve metric for each predictive model, ranging from 0.76 ± 0.1 to 0.77 ± 0.1 for RRF and LASSO models, respectively. The features selected by each ML algorithm in order of importance in AKI prediction are displayed in Supplementary Figure 5C.

Altogether, this sensitivity analysis strengthens both the use of nonlinear fitting between numerical predictors and risk of AKI, as well as the choice of the predictors.

### Identification of AKI phenotypes

#### Clustering of AKI patients according to their risk factors pattern

**Figure 2:**
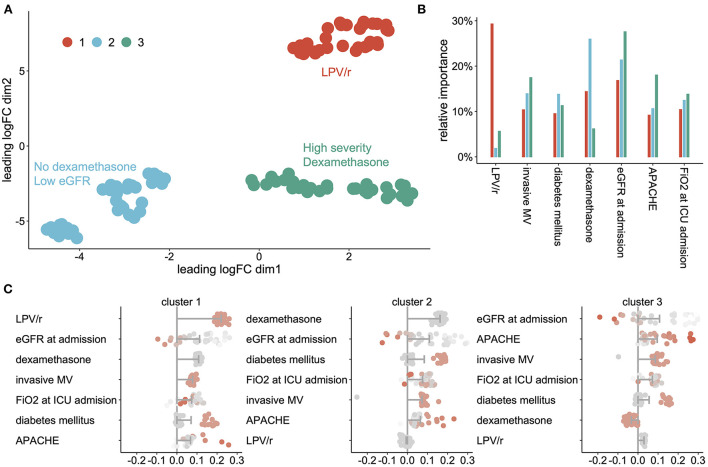
AKI phenotypes' **(A)** scatterplot showing the cluster of AKI patients projected on the UMAP, **(B)** relative importance of each variable across clusters, **(C)** shapley Additive Explanation (SHAP) values for each cluster of AKI patients, sorted by impact on AKI prediction. Bars represent the mean impact of each AKI associated factor for each cluster and dots represent individual patients. LPV/r Lopinavir/Ritonavir, DM Diabetes Mellitus; DXM Dexamethasone; MV Mechanical Ventilation.

Among the 99 AKI patients, we were able to identify three clusters, each of them expressing a specific pattern of AKI-related factors (Figure 2A). The relative importance of each variable across clusters is shown in Figure 2B. Use of LPV/r, dexamethasone and eGFR/APACHE score were the most discriminant factors of cluster 1, 2, and 3, respectively. Figure 2C shows the predictors, in order of importance, that defined each cluster. Cluster 1 was characterized by AKI associated with the use of LPV/r; cluster 2 involved patients with lower baseline eGFR who did not receive dexamethasone; cluster 3 included the most severe patients with low baseline eGFR who however were receiving dexamethasone.

#### Sensitivity analyses

SVM models validated the separation of the three clusters form the others with areas under ROC curves in the test dataset equal to 1.0 ± 0 for each cluster.

### Clinical characteristics and outcomes of the three AKI phenotypes

**Figure 3:**
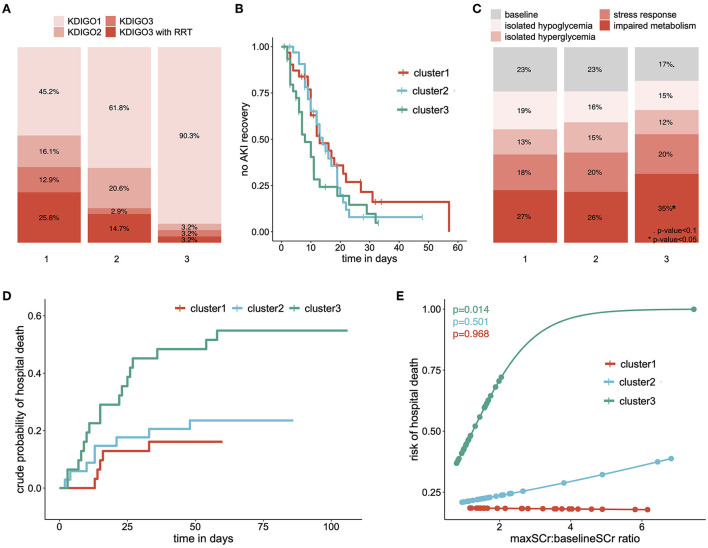
Clinical outcomes of each cluster: **(A)** repartition of AKI severity among clusters, according to the KDIGO criteria **(B)** survival curve showing the proportion of patients who did not experience AKI recovery, over time and among clusters, **(C)** relative time spent in each metabolic pattern according to clusters of AKI patients, **(D)** cumulative incidence curve of the hospital mortality, stratified on clusters of AKI patients and **(E)** predicted risk of hospital death according to the ratio of maximal and baseline serum creatinine level among clusters. eGFR estimated Glomerular Filtration Rate; RRT Renal Replacement Therapy. *p*-value < 0.1; *p-value < 0.05; **p-value < 0.001; ***p-value < 0.0001.

Patients from cluster 3 developed less severe AKI than patients from cluster 1 and 2 (6% [0–13] vs. 28% (15–38) of KDIGO3 AKI, *p* = 0.009) and less frequently received RRT (3% [0–6] vs. 20% (9–29), *p* = 0.02) (Figure 3A). They also displayed a higher recovery rate (HR = 1.6 for AKI recovery, 95% CI [1.0; 2.7], *p* = 0.05, Figure 3B). In addition, patients from cluster 3 also displayed a distinct metabolic profile, expressing the impaired metabolism profile at a higher rate (35% (26–43) vs. 27% (22–31) *p* = 0.04, Figure 3C), and had a higher hospital mortality (55% [39-71] vs. 20% (11–29) *p* < 0.001, Figure 3D). Finally, only patients from cluster 3 exhibit a significant positive association between AKI severity and risk of hospital mortality (Figure 3E).

Altogether, this analytic procedure allowed us to identify 3 clusters of AKI patients, each of them expressing a specific pattern of factors associated with AKI. These patients also displayed different clinical characteristics, including different AKI severity, mortality and recovery.

## Discussion

The current definition of AKI is limited as it provides no information on AKI etiology, prognosis, molecular pathways, or responses to treatment (36). Here we identified phenotypes of AKI patients based on their pattern of AKI associated factors, with distinct characteristics and outcomes.

We first identified factors associated with AKI development. When considering COVID-19 specific therapy, we found LPV/r and dexamethasone to be, respectively positively and negatively correlated to AKI development, in accordance with other groups (37–41). We also reported well described AKI risk factors, such as diabetes mellitus and baseline eGFR (42, 43). Finally, we identified FiO2 and a need for mechanical ventilation at ICU admission. While high FiO2 may only reflect disease severity, mechanical ventilation could be causative. Previous studies already reported an association between mechanical ventilation requirement and AKI occurrence in COVID-19 patients (44, 45). Animal data has described renal hemodynamic alterations during invasive mechanical ventilation well (46, 47). In particular, the PEEP level could play an ambivalent role, with beneficial effects like lung volume recruitment at the cost of an increase in central venous pressures (CVP) (48). Elevated CVP has been associated with reduced renal blood flow, glomerular filtration rate and urine output (49), as well as activation of sympathetic nervous system and renin-angiotensin-aldosterone system and suppression of the atrial natriuretic peptide, all resulting in kidney injury (49–53).

In our cohort of AKI COVID-19 patients, our pipeline was able to identify three clusters of patients. At the renal level, while all patients met the criteria for AKI, each cluster displayed a distinct phenotype in terms of KDIGO stage and AKI recovery. In particular, cluster 1 involving patients receiving LPV/r was characterized by severe AKI with 26% of patients requiring renal replacement therapy while cluster 3 includes only 3.2% of dialyzed patients (p=0.008). However, only patients from cluster 3 displayed the commonly accepted association between AKI severity and mortality. These patients also exhibited a higher rate of impaired metabolism pattern and a greater severity (Supplementary Table 2), in line with our previous results (18). This may suggest that patients from clusters 1 and 2 developed a distinct form of AKI.

Altogether, these three phenotypes may reflect distinct pathophysiological mechanisms of AKI development that does not result in differences in serum creatinine levels.

Beyond these results, this study introduces a pipeline of analyses, which is able to phenotype AKI patients according to their pattern of risk factors, with several innovative features. First, while most of the studies identified AKI risk factors through logistic regression (45, 54), we used a generalized additive model with regression splines to capture nonlinear associations between AKI and potential risk factors. This method allowed us to identify factors that would have remained otherwise unnoticed with the traditional approach (i.e., baseline eGFR, APACHE score and FiO2 at ICU admission). Furthermore, we calculated the absolute importance of each risk factor in estimating the probability of AKI for each patient. We thus obtained a pattern of risk factors for each patient that may reflect a specific pathophysiological mechanism. Existing studies on AKI phenotyping have either used supervised clustering, mostly on clinical traits (13, 14), or unsupervised clustering based on recorded clinical or biological data (15–17). Finally, we did not apply the clustering algorithm on the raw dataset as did other groups (15–17), but rather on a dimensionally reduced space; a strategy that has been shown to improve the clustering performance (33).

Our study has some limitations. The first is that the study was single-centered which limits the extent of our results. The second is that being a retrospective study, procedures and therapeutic strategies may have changed during the study period. Lastly, because of the low sample size and the use of a flexible model (i.e., the generalize additive model), identification of factors associated with AKI may be spurious. However, the same factors were independently found by three unsupervised machine learning models with built-in feature selection. Similarly, a non-linear relation was also confirmed using the LOESS regression.

In summary, we have developed a new pipeline of analyses which identified 3 subgroups of AKI patients with distinct renal features and outcomes that may be related to specific pathophysiological mechanisms. This pipeline is generalizable pipeline and may be applied to various datasets to identify patients with different outcomes and therapeutic sensitivity.

## Data availability statement

The raw data supporting the conclusions of this article will be made available by the authors, without undue reservation.

## Ethics statement

The studies involving human participants were reviewed and approved by Commission Cantonale d'Ethique de la Recherche. Written informed consent for participation was not required for this study in accordance with the national legislation and the institutional requirements.

## Author contributions

Conceptualization and supervision: DL. Methodology and formal analysis: DL and GC. Validation: DL, GC, and JP. Data curation: DL, FS, EM, and CL. Writing—original draft preparation: DL, FS, and EM. Writing—review and editing: DL, GC, CL, SS, and JP. All authors contributed to the article and approved the submitted version.

## Funding

DL is supported by two young researcher grants from the Geneva University Hospitals (PRD 5-2020-I and PRD 4-2021-II) and by a grant from the Ernst and Lucie Schmidheiny Foundation. Open access funding was provided by the University of Geneva.

## Conflict of interest

The authors declare that the research was conducted in the absence of any commercial or financial relationships that could be construed as a potential conflict of interest.

## Publisher's note

All claims expressed in this article are solely those of the authors and do not necessarily represent those of their affiliated organizations, or those of the publisher, the editors and the reviewers. Any product that may be evaluated in this article, or claim that may be made by its manufacturer, is not guaranteed or endorsed by the publisher.
