# Erratum: Unsupervised clustering reveals phenotypes of AKI in ICU COVID-19 patients

**DOI:** 10.3389/fmed.2023.1172589

**Published:** 2023-03-07

**Authors:** Frontiers Production Office

**Affiliations:** Frontiers Media SA, Lausanne, Switzerland

**Keywords:** AKI, clustering, machine learning, COVID-19, critical care

An omission to the funding section of the original article was made in error. The following sentence has been added: “Open access funding was provided by the University of Geneva.”

The original article has been updated.

