# Supplementary material for: Unsupervised clustering reveals phenotypes of AKI in ICU COVID-19 patients
Source: Front Med (Lausanne). 2022 Oct 5;9:980160. doi: 10.3389/fmed.2022.980160 (PMC9579431; doi:10.3389/fmed.2022.980160)
Supplement: Supplementary file 1 [file Data_Sheet_1.PDF]

Additional File 1

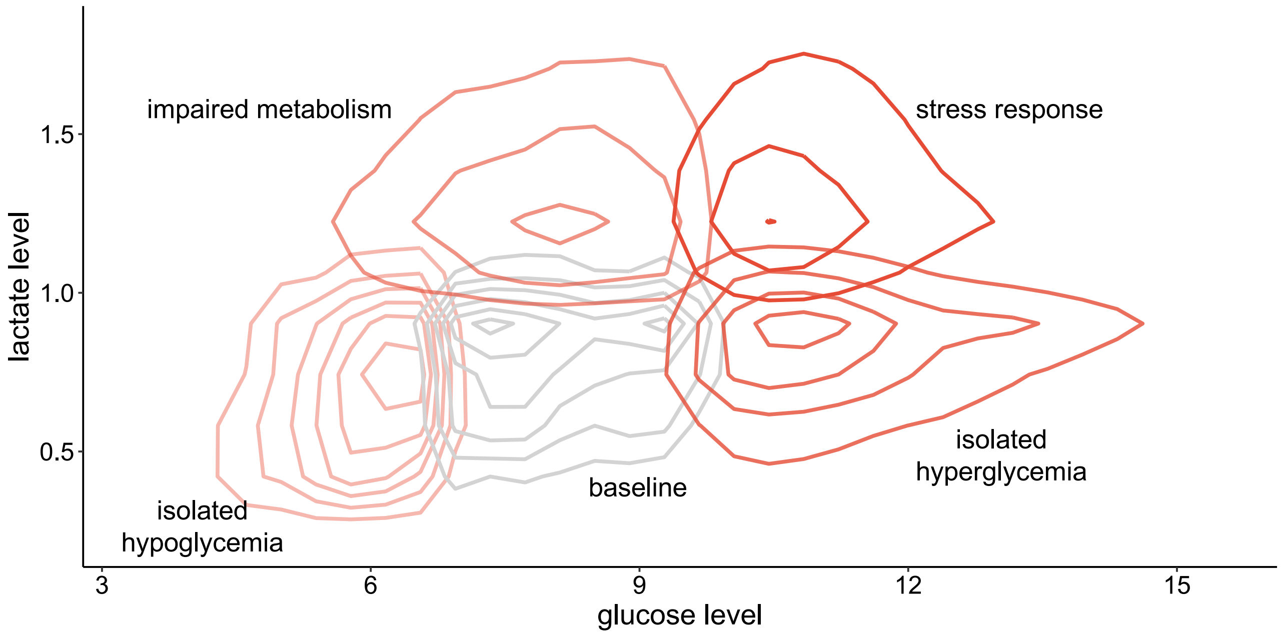

definition of the 5 metabolic patterns: density plot showing lactate and glucose levels in mmol/L according to the five metabolic patterns defined.

Additional File 2

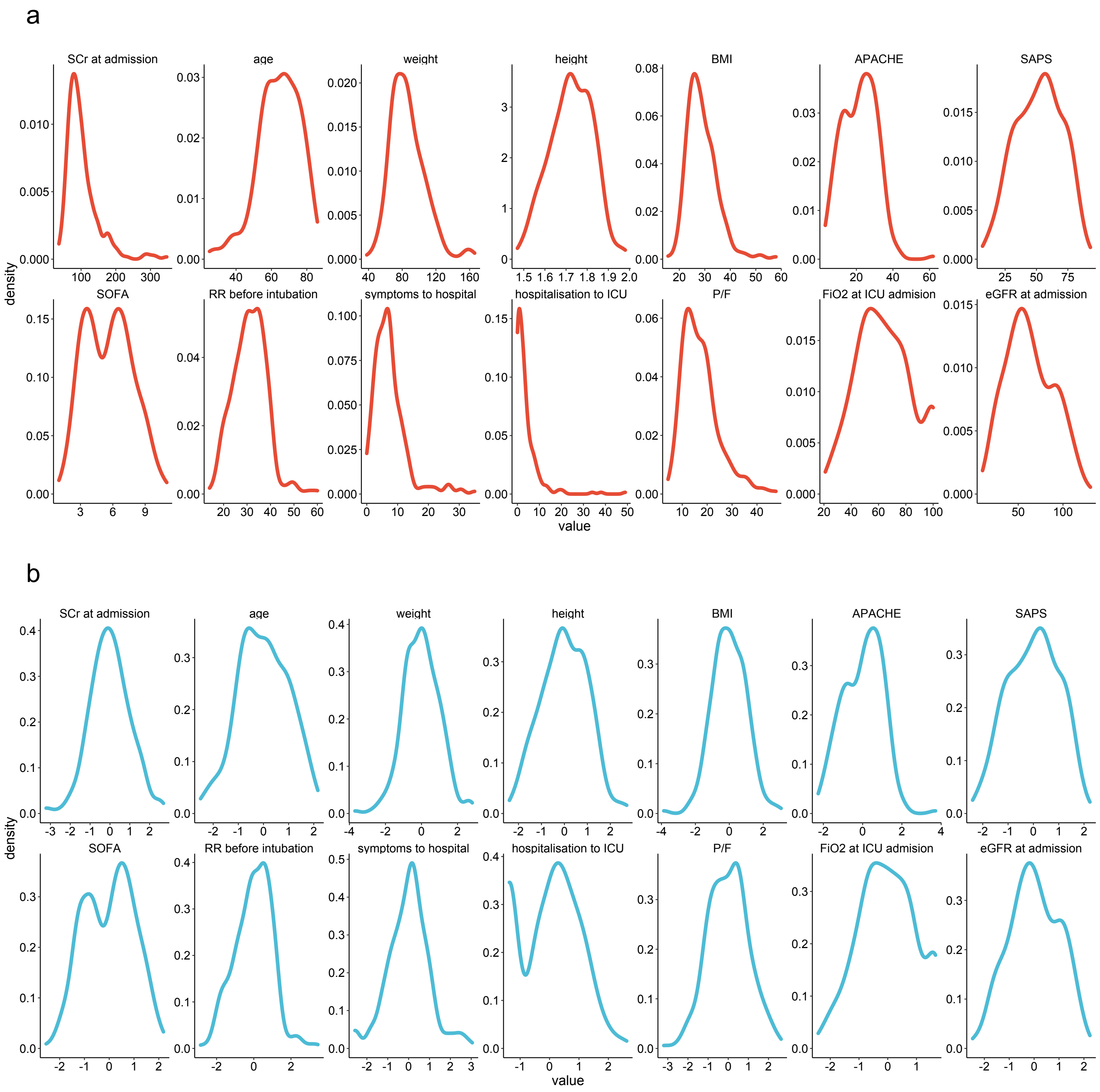

data transformation: distribution of the numerical variables before (a) and after (b) scaling, centering and Yeo-Johnson transformation. SCr Serum Creatinine; BMI Body Mass Index; RR Respiratory Rate; P/F PaO2/FiO2 ratio; PCT Procalcitonin; CRP C-Reactive Protein; WBC White Blood Cells; NLR Neutrophils to Lymphocytes Ratio; PP Prone Positioning; NMBA Neuro Muscular Blocking Agents.

Additional File 3

a

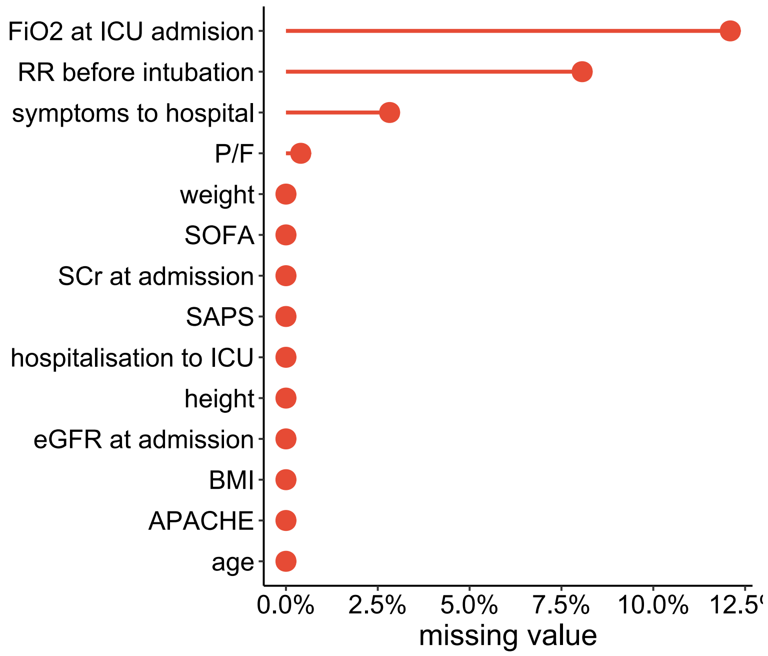

b

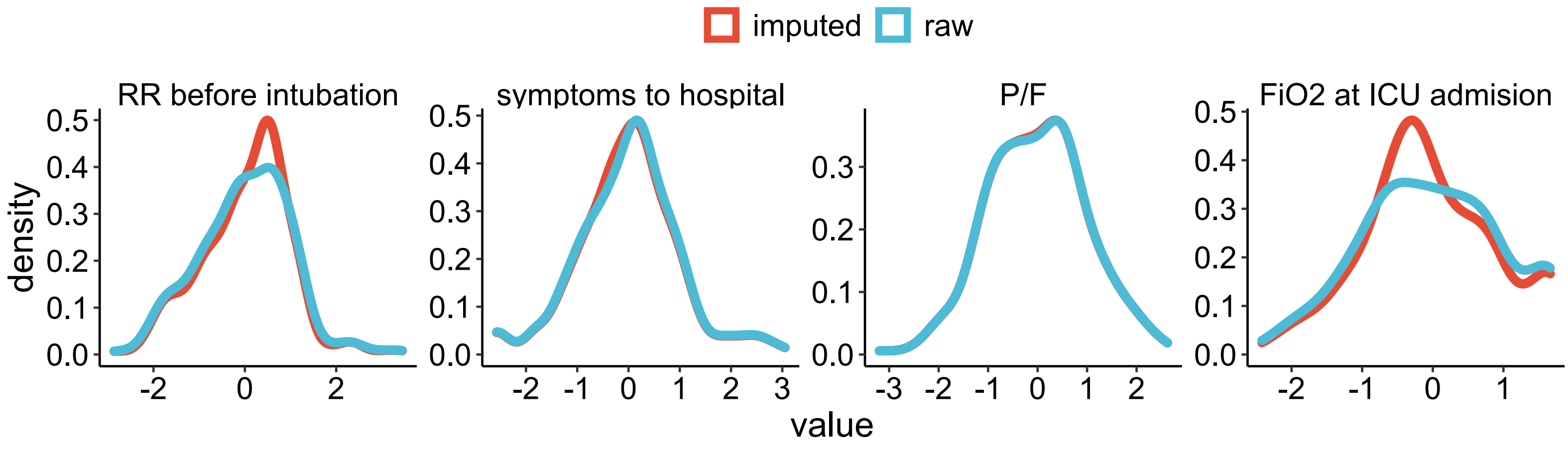

handling of missing value: a) missing value rate for each recorded variable and b) distribution of the variable before (blue) and after (red) data imputation. SCr Serum Creatinine; BMI Body Mass Index; RR Respiratory Rate; P/F PaO2/FiO2 ratio; PCT Procalcitonin; CRP C-Reactive Protein; WBC White Blood Cells; NLR Neutrophils to Lymphocytes Ratio; PP Prone Positioning; NMBA Neuro Muscular Blocking Agents.

Additional File 4

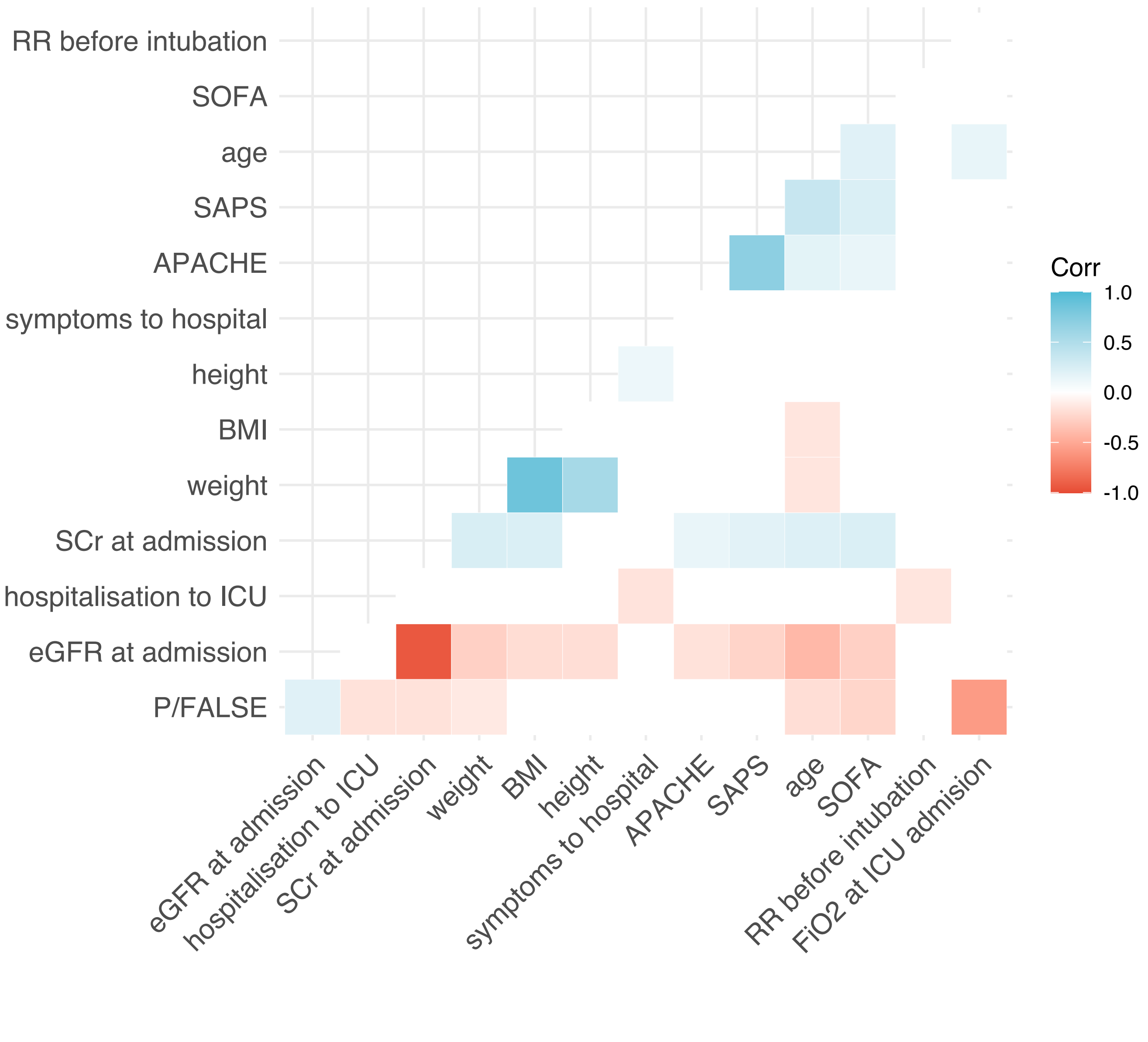

correlated variables: correlation matrix showing the correlation between each recorded variable. Blank cases mean non-significant correlation (p-value>0.05). BMI Body Mass Index; NO Nitric Oxide; NMBA Neuro Muscular Blocking Agents; WBC White Blood Cells; NLR Neutrophils to Lymphocytes Ratio; PCT Procalcitonin; CRP C-Reactive Protein; eGFR estimated Glomerular Filtration Rate; P/F PaO2/FiO2 ratio; SCr Serum Creatinine; RAAS Renin Angiotensin Aldosterone System blockers; NSAIDs Non-Steroidal Anti-inflammatory Drugs; COPD Chronic Obstructive Pulmonary Disease; LPV/r Lopinavir/Ritonavir, MV Mechanical Ventilation

Additional File 5

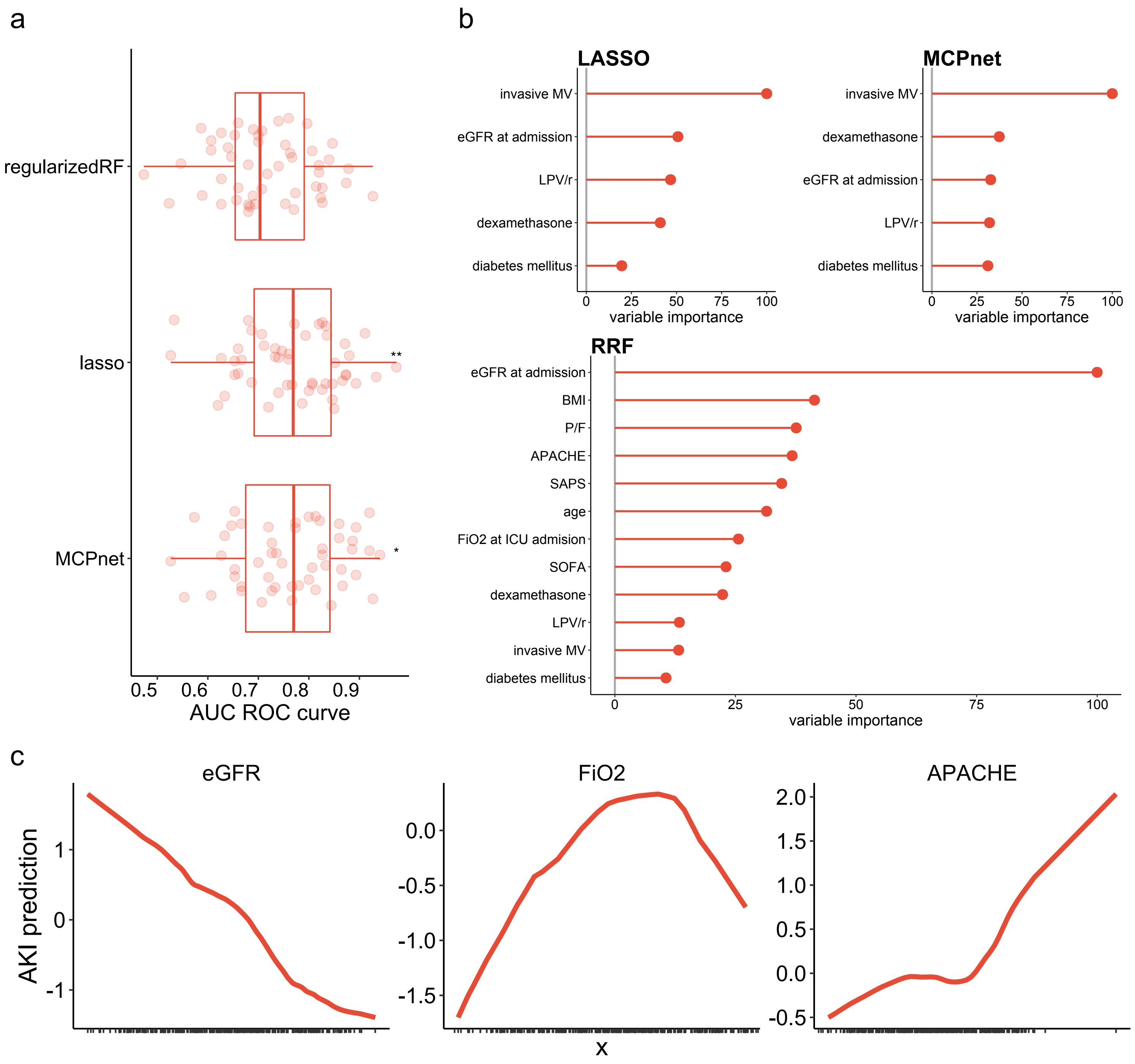

model validation: a) boxplot showing the distribution of the area under the receiver operating characteristic curve for each machine learning algorithm trained to predict AKI, b) importance of the selected variables for AKI prediction for each machine learning method and c) partial dependence plots, showing the effect of baseline eGFR, APACHE score and FiO2 at ICU admission on the risk of AKI, extracted from the generalized additive model with LOESS fitting. Tick values on the x-axis shows the feature distribution in the dataset. LPV/r Lopinavir/Ritonavir; DXM Dexamethasone; eGFR estimated Glomerular Filtration Rate; BMI Body Mass index; P/F PaO2/FiO2 ratio; RR Respiratory Rate.
