# Supplementary material for: Unsupervised clustering reveals phenotypes of AKI in ICU COVID-19 patients
Source: Front Med (Lausanne). 2022 Oct 5;9:980160. doi: 10.3389/fmed.2022.980160 (PMC9579431; doi:10.3389/fmed.2022.980160)
Supplement: Supplementary file 2 [file Table_1.docx]

**Additional Table 1:** variables selected in the final generalized additive mode for AKI prediction with corresponding pvalue. LPV/r Lopinavir/Ritonavir; PCT Procalcitonin; NMBA Neuromuscular Blocking Agent; eGFR estimated Glomerular Filtration Rate

| **variable** | **pvalue** |
| --- | --- |
| LPV/r | 0.032 |
| invasive MV | 0.007 |
| diabetes mellitus | 0.013 |
| dexamethasone | 0.004 |
| s(GFRfirst) | <0.001 |
| s(APACHE) | 0.194 |
| s(FiO2_admission) | 0.042 |
