# Supplementary material for: Unsupervised clustering reveals phenotypes of AKI in ICU COVID-19 patients
Source: Front Med (Lausanne). 2022 Oct 5;9:980160. doi: 10.3389/fmed.2022.980160 (PMC9579431; doi:10.3389/fmed.2022.980160)
Supplement: Supplementary file 3 [file Table_2.docx]

**Additional Table 2**: baseline characteristics at ICU admission among cluster. WBC White Blood Cell count; NLR Neutrophil to Lymphocyte Ratio

|  | Cluster 1 (N=32) | Cluster 2 (N=35) | Cluster 3 (N=32) | p value |
| --- | --- | --- | --- | --- |
| **APACHE** |  |  |  |  |
| Median (Q1, Q3) | 17.5 (11.8, 25.2) | 23.0 (18.0, 29.5) | 29.0 (20.5, 33.2) | 0.003 |
| **SAPS** |  |  |  |  |
| Median (Q1, Q3) | 46.0 (30.5, 63.2) | 55.0 (43.5, 63.0) | 61.0 (45.5, 71.8) | 0.034 |
| **WBC** |  |  |  |  |
| Median (Q1, Q3) | 7.0 (5.1, 9.1) | 9.7 (6.8, 10.6) | 12.4 (9.8, 15.8) | < 0.001 |
| **NLR** |  |  |  |  |
| Median (Q1, Q3) | 12.7 (6.6, 20.2) | 15.4 (5.5, 19.8) | 20.2 (14.9, 43.7) | 0.004 |
| **thrombocytes** |  |  |  |  |
| Median (Q1, Q3) | 182.0 (140.0, 231.0) | 209.0 (150.0, 250.0) | 267.5 (200.5, 342.0) | 0.001 |
| **lactate** |  |  |  |  |
| Median (Q1, Q3) | 0.8 (0.7, 1.1) | 0.9 (0.7, 1.2) | 1.4 (1.3, 1.9) | < 0.001 |
| **bilirubin** |  |  |  |  |
| Median (Q1, Q3) | 11.5 (7.2, 18.5) | 8.0 (6.0, 10.0) | 12.0 (7.8, 16.2) | 0.021 |
